# The effectiveness of instrument-assisted soft tissue mobilization on range of motion: a meta-analysis

**DOI:** 10.1186/s12891-024-07452-8

**Published:** 2024-04-23

**Authors:** Sien Tang, Li Sheng, Jinming Xia, Bing Xu, Peiyong Jin

**Affiliations:** The Fourth Rehabilitation Hospital of Shanghai, No. 995 Kangding Road, Jing’an District, Shanghai, 200000 China

**Keywords:** Instrument-assisted soft tissue mobilization, Range of motion, Meta-analysis

## Abstract

**Background:**

To evaluate the effectiveness of instrument-assisted soft tissue mobilization (IASTM) on range of motion (ROM).

**Methods:**

We performed a literature search of the PubMed, Embase, Web of Science, and Cochrane Library databases from inception to December 23, 2023. Randomized controlled trials that compared treatment groups receiving IASTM to controls or IASTM plus another treatment(s) to other treatment(s) among healthy individuals with or without ROM deficits, or patients with musculoskeletal disorders were included. The Cochrane risk of bias tool was used to assess the risk of bias.

**Results:**

Nine trials including 450 participants were included in the quantitative analysis. The IASTM was effective in improving ROM in degree in healthy individuals with ROM deficits and patients with musculoskeletal disorders (*n*=4) (MD = 4.94, 95% CI: 3.29 to 6.60), and in healthy individuals without ROM deficits (*n*=4) (MD = 2.32, 95% CI: 1.30 to 3.34), but failed to improve ROM in centimeter in healthy individuals with ROM deficits (*n*=1) (MD = 0.39, 95% CI: -1.34 to 2.11, *p*=0.66, I^2^ = 88%).

**Conclusions:**

IASTM can improve ROM in degree in healthy individuals with or without ROM deficits, or in patients with musculoskeletal disorders (with very low to low certainty).

**Trial registration:**

The PROSPERO registration ID is CRD42023425200.

**Supplementary Information:**

The online version contains supplementary material available at 10.1186/s12891-024-07452-8.

## Background

Musculoskeletal disorders are among the most common types of human diseases and can affect all parts of the body [1, 2]. Surveys have revealed that musculoskeletal disorders affect more than a billion people worldwide, and are showing an increasing trend annually [1, 2]. Musculoskeletal disorders not only induce pain and joint adhesions that disrupt normal body movement but also have the potential to trigger mental health issues such as depression and stress [3, 4]. Range of motion (ROM) deficits are a critical predisposing factor and clinical manifestation of musculoskeletal disorders [4–6]. The effects and symptoms of ROM deficits are not limited to the joints and muscles directly affected, but may even involve other areas [7–9]. Consequently, improving ROM is seen as a crucial step in both the prevention and treatment of these conditions.

There are different ways of improving ROM, such as PRP and PRF injections, biofeedback, medications, physiotherapy, and surgery [6, 10–13]. Among these, physiotherapy has the widest range of applications. It can be used not only to treat patients, but also to treat healthy people [6, 14]. Currently, there are various methods used in physiotherapy that can improve ROM, such as stretching, relaxation and mobilization [6, 14]. Among these methods, instrument-assisted soft tissue mobilization (IASTM) is gaining popularity [15]. Soft tissues should be released based upon the principles of cross-friction massage and specially designed manual instruments [16, 17].

However, the efficacy of IASTM on ROM has not been consistently supported by clinical studies [18–21]. It is necessary to review these studies to evaluate the effectiveness of IASTM. To date, two meta-analyses, both of which were conducted by the same team, have concluded that the evidence does not support that IASTM could improve ROM [22, 23]. However, both of these studies have important limitations. Both studies presented analyses of individuals with or without ROM deficits simultaneously, which may underestimate the effectiveness of IASTM. They also compared the effects of IASTM with those of other treatments or placebo, which may have produced incorrect results. In addition, the use of minimal clinically important difference to assess the effectiveness of treatment is misleading when healthy individuals without ROM deficits are included. Therefore, it is reasonable to re-assess the effectiveness of IASTM on ROM. The aim of this meta-analysis was to assess the effect of IASTM on ROM in healthy individuals with or without ROM deficits, or patients with musculoskeletal disorders.

## Methods

This meta-analysis followed the updated guidelines of the Preferred Reporting Items for Systematic Reviews and Meta-Analyses (PRISMA, 2020) and has been registered on the PROSPERO website (RegNo. : CRD42023425200) [24].

### Eligibility criteria

Studies were included if they met the following criteria: (1) were randomized controlled clinical trials; (2) were healthy individuals with or without ROM deficits, or patients with musculoskeletal disorders; (3) compared IASTM alone to control or IASTM plus another treatment(s) to other treatment(s); and (4) had an outcome of ROM. We had no language restrictions.

Studies were excluded if the following criteria were met: (1) no mention of randomization in the text; (2) the described randomization was nonrandom; or (3) lacked outcome data of interest.

### Information sources

Since instrument-assisted soft tissue mobilization (IASTM) is not a medical subject heading (MeSH), we expanded the entry terms to cover both instrument-assisted and manual mobilization. We searched the PubMed, Embase, Web of Science, and Cochrane Library databases from inception to December 23, 2023, by using the syntax shown in Additional file 1. The references of published systematic reviews were examined to ensure the retrieval of all available studies that had been included in the meta-analysis.

### Study selection

Two researchers (S. Tang and L. Sheng) independently carried out the study selection: (1) all retrieved studies were imported into EndNote 21 software (Ceverbridge Analytics, Philadelphia, PA, USA), and duplicates were removed; (2) clearly irrelevant studies were judged by the title and abstract and excluded; and (3) the full texts of relevant studies were then retrieved, and the final included studies met both the inclusion and exclusion criteria. In cases of disagreement, a consensus was reached through discussion.

### Data extraction

We designed a pilot Excel form (by S. Tang) to independently extract data from five representative studies by two researchers (S. Tang and L. Sheng). The final Excel form was developed from the pilot form following discussion and modification. These two researchers independently extracted the data from all the included studies. The extracted data were cross-checked, and in the case of any disagreements, a consensus was reached by recreating the process of selecting the study and calculating the data. Information on the study identification and principles of the PICOS (participant, intervention, control, outcome and study design) was extracted. The outcome data of interest were the mean difference (MD) and its standard deviation (SD) (or its 95% confidence interval, 95% CI) of ROM from baseline in two parallel groups.

The data for analysis were as follows: (1) for subgroup data from multiarm trials, the sample size was split by the number of arms; (2) for studies in which multiple measurements were used to assess the same outcome, only the most reliable measurement was used; (3) for studies in which multiple outcomes (except inversion and eversion of the ankle due to the small data) were used for the relevant outcomes, and the sample size was averaged based on the number of outcomes; (4) for studies in which only the outcome at the end of the treatment was used but not the intermediate measurements or those during follow-up were used; and (5) for studies in which the MD and SD from baseline were not reported, we converted from the CI and standard errors (SE), when available, by using the calculator provided in RevMan 5.4 (the Cochrane Collaboration, London, UK). If no outcome data were available, we contacted the authors through emails for their research results. If data from the study authors were unavailable, the data were estimated by using the data from other studies. The following formulas were used for extrapolation [25]:

1 $${\text{R=}}\frac{{\text{SDbaseline}}^{2}{\text{+SDfinal}}^{2}-{\text{SDchange}}^{2}}{\text{2*SDbaseline*SDfinal}}$$

2 $${\text{SDchange=}}\sqrt{{\text{SDbaseline}}^{2}{\text{+SDfinal}}^{2}{\text{-2*R*SDbaseline*SDfinal}}}$$

### Assessment of the risk of bias

Two researchers (S. Tang and J. Xia) independently assessed the risk of bias of the included studies (see Additional file 2). In cases of disagreement, a third researcher (L. Sheng) participated in the discussion and reached a consensus. The Cochrane risk of bias tool was used to assess the risk of bias. Each of the seven risk of bias domains was rated as “low”, “unclear”, or “high” [26]. The other bias and overall risk of the study were assessed using the method employed by Goris et al. [23] The other bias was defined as studies published in suspected predatory journals, as identified by Manca et al. [27] The overall risk of bias was as follows: if all risk of bias was rated as low, then the study was rated as low risk; if at least one of the risk of bias was rated as unclear, then the study was rated as unclear risk; and if at least one of the risk of bias was rated as high, then the study was rated as high risk [23]. Considering the nature of the IASTM intervention, if a study merely had a high risk of bias due to the blinding of participants and personnel, the study was not rated as high risk. Instead, it was rated as either low risk (if the remaining six domains were rated as low risk) or unclear risk (if one or more of the remaining six domains were rated as unclear risk) [22, 23].

### Statistical analysis

The data were analyzed by using Review Manager 5.4 (the Cochrane Collaboration, London, UK) and Stata 14 (StataCorp LLC, Texas, USA). Heterogeneity was estimated by using the Cochran Q and I^2^ indices. If *P* ≥ 0.1 and I^2^ ≤ 50%, indicating low heterogeneity, the fixed effects model was used; if *P* < 0.1 and I^2^> 50%, indicating significant heterogeneity, the random-effects model was applied [25]. The mean difference and 95% CI are reported for the synthesized data in the forest plot. Subgroup analyses were conducted according to intervention methods (combined therapies or IASTM alone). Due to the limited number of studies included, publication bias was not evaluated [25]. Sensitivity analyses were performed using leave-one-out tests to confirm the stability of the results [25].

## Results

### Study selection

**Fig. 1:**
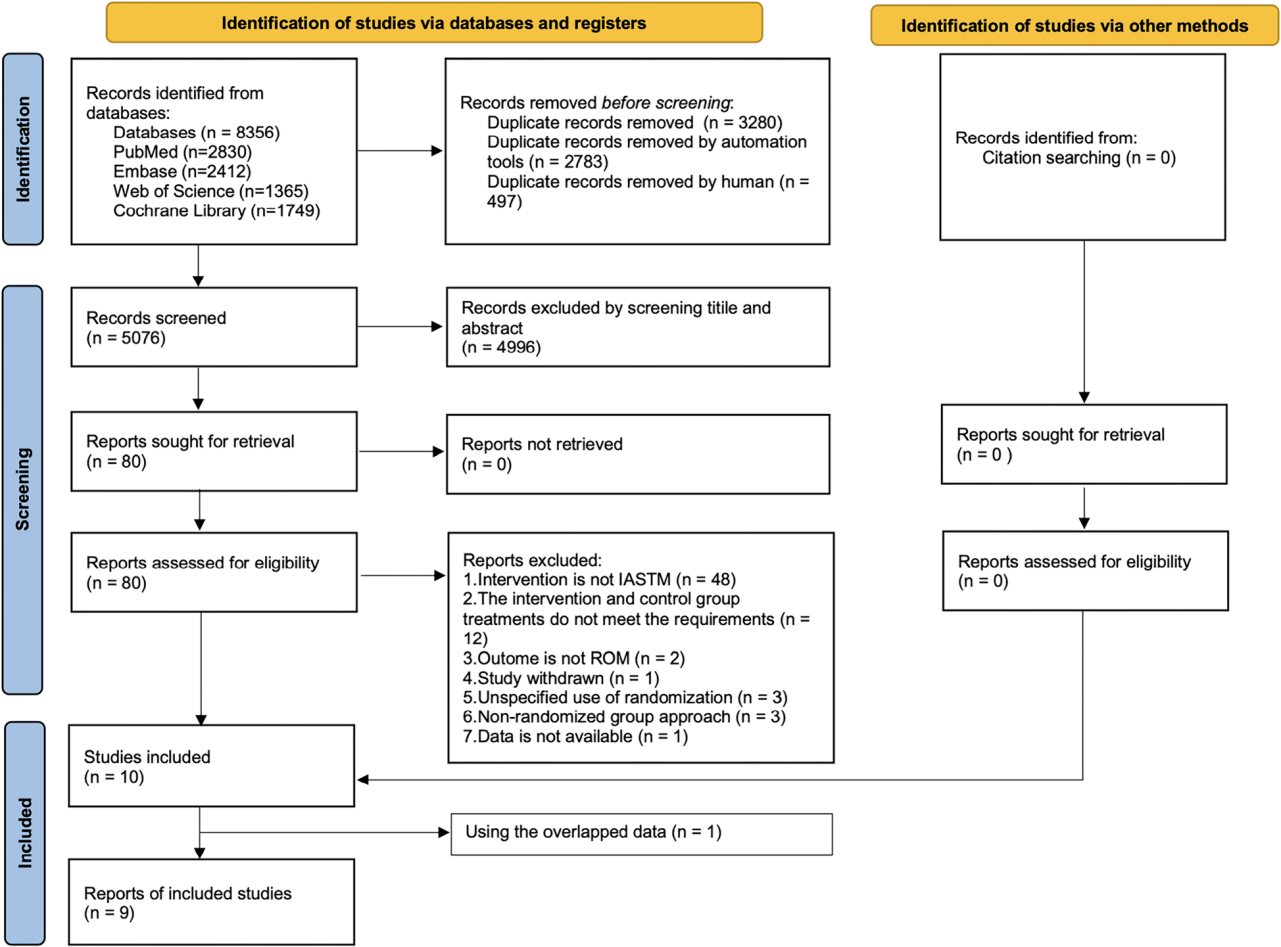
Study selection process

A total of 8356 articles were identified: 2830 from PubMed, 2412 from Embase, 1365 from Web of Science, and 1749 from the Cochrane Library. No additional studies were identified from other sources. After removing duplications, 5076 articles remained, and 4996 clearly irrelevant studies were excluded based on the titles and abstracts. The full texts of the remaining 80 articles were retrieved and read carefully. Ultimately, a total of 10 studies that met both the inclusion and exclusion criteria were included [28–37]. Two different studies published by the same author used overlapping data [31, 32], we excluded the study published in 2017 [32] (we thought the data in this piece extended from the 2015 study [31, 32]) and analyzed the data from the remaining 9 studies (Fig. 1) [28–31, 33–37].

### Study characteristics

The 9 included studies were published between 2012 and 2022 and involved a total of 522 participants [28–31, 33–37]. The age of the study participants was not described in one of the studies [35], whereas the remaining 8 studies had an average age of 27.17 ± 10.96 years [28–31, 33, 34, 36, 37]. Two studies did not provide information about the gender of the participants [30, 34]. Among the remaining 7 studies, the proportion of male participants was 61.67% [28, 29, 31, 33, 35–37]. Regarding study characteristics, 4 studies focused on healthy individuals without ROM deficits [30, 33, 34, 36], 3 studies focused on healthy individuals with ROM deficits [31, 35, 37], and 2 studies included patients with musculoskeletal disorders [28, 29]. Additionally, 6 studies treated only one session [30, 31, 33–36], while 3 studies treated multiple sessions [28, 29, 37]. Furthermore, only IASTM was used in 2 studies [33, 34], combined therapies were used in 6 studies [28–31, 36, 37], and one study included both alone and in combination [35]. Eight studies of ROM used degrees as a unit of measurement [28–31, 33, 34, 36, 37], while 1 study used centimeters (assessed by the lunge test) [35]. A summary of the 9 studies is shown in Table 1 (at the end of the paper).

**Table 1:** Summary of included studies

study	Participant age ± SD (y) / males (%)	Groups / N	Outcome	IASTM Duration
Abdel-aal et al (2021) [28]	Patients with cervicogenic headache 41.69 ± 4.89 / 38.3 %	Intervention group / 30: exercise program + IASTM Control group / 30: exercise program	Cervical ROM: flexion, extension, left lateral flexion, right lateral flexion, left rotation, right rotation	approximately 3 min per time, 3 times per week for 4 weeks
Aggarwal et al(2021) [29]	Patients of shoulder adhesive capsulitis 49.4 ± 8.13 / 23.3 %	Intervention group / 15: conventional treatment + IASTM Control group / 15: conventional treatment	Passive and active shoulder ROM: flexion, extension, abduction, internal rotation, external rotation	2 min per time, 3 times per week for 4 weeks
Angelopoulos et al(2021) [30]	Healthy amateur overhead athletes (dominant shoulders) 23.03 ± 1.89 / no description	Intervention group / 20: IASTM + kinetic flossing Control group / 20: kinetic flossing IASTM group / 20: IASTM KT group / 20: kinesiology taping	Passive shoulder ROM: internal rotation, external rotation	6 min per time, one time
Bailey et al(2015) [31]	Asymptomatic baseball players with ROM deficits 19 ± 2 / 100 %	Intervention group / 30: IASTM + self-stretching Control group / 30: self-stretching	Passive shoulder ROM: horizontal adduction, internal rotation, external rotation	2 min per time, one time
Ikeda et al(2019) [33]	Health individuals (right leg) 24 ± 4 / 78.6 %	Intervention group / 7: IASTM Control group / 7: no treatment	Passive ankle ROM: dorsiflexion	5 min per time, one time
Laudner et al(2014) [34]	Asymptomatic collegiate baseball players (their throwing arm) 20.1 ± 1.2 / no description	Intervention group / 17: IASTM Control group / 18: no treatment	Passive shoulder ROM: horizontal adduction, internal rotation	40 s per time, one time
Lehr et al(2022) [35]	Healthy collegiate athletes (the more restricted leg) No description / 66 %	Combine group / 34: IASTM + MWM Intervention group / 36: IASTM Control group / 33: no treatment MWM group / 44: MWM	Passive ankle ROM: dorsiflexion	2 min per time, one time
Rowlett et al(2019) [36]	Health individuals 25.8 ± 6.7 / 36.7 %	Intervention group / 20: warm-up + IASTM	Passive ankle ROM: dorsiflexion	2 min per time, one time
		Stretch group / 20: warm-up +stretching Control group / 20: warm-up		
Schaefer & Sandrey(2012) [37]	Healthy individuals with a history of chronic ankle instability 17.7 ± 1.9 / 86.1 %	Intervention group / 13: warm up + IASTM + balance training Sham group / 12: warm up + sham IASTM + balance training Control group / 11: warm up + balance training	Active ankle ROM: dorsiflexion, flexion, inversion, evrsion	8 min per time, 2 times per week for 4 weeks

*IASTM* Instrument-assisted soft-tissue mobilization, *KT* Kinesiology taping, *MWM* Mobilization with movement, *ROM* Range of motion, *SD* Standard deviation

### Risk of bias assessment

**Fig. 2:**
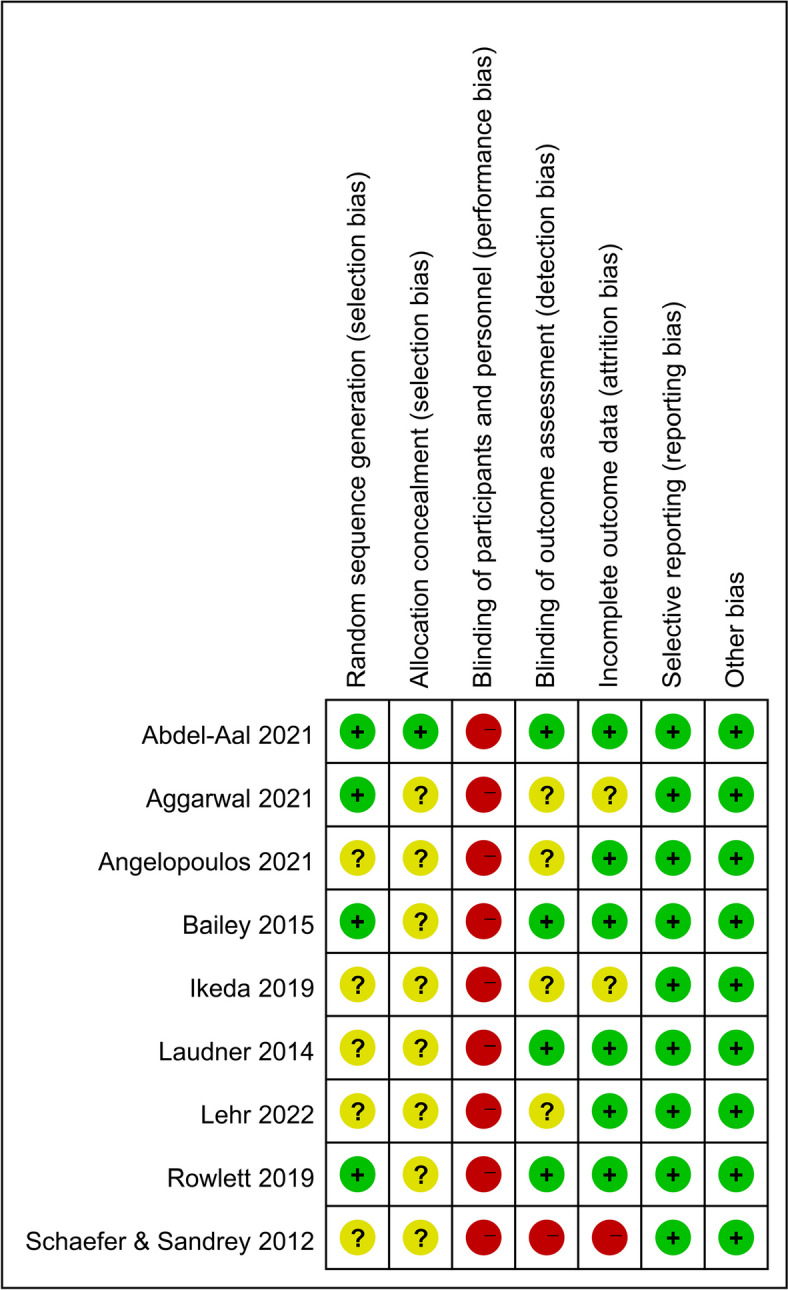
Risk of bias summary

The risk of bias assessment of the 9 studies is presented in Fig. 2. From the overall risk of the study, one study was rated as low risk [28], seven studies were rated as unclear risk [29–31, 33–36], and one study was rated as high risk [37].

### Outcomes

#### Effect of IASTM on ROM in healthy individuals with ROM deficits and patients with musculoskeletal disorders (in degree)

**Fig. 3:**
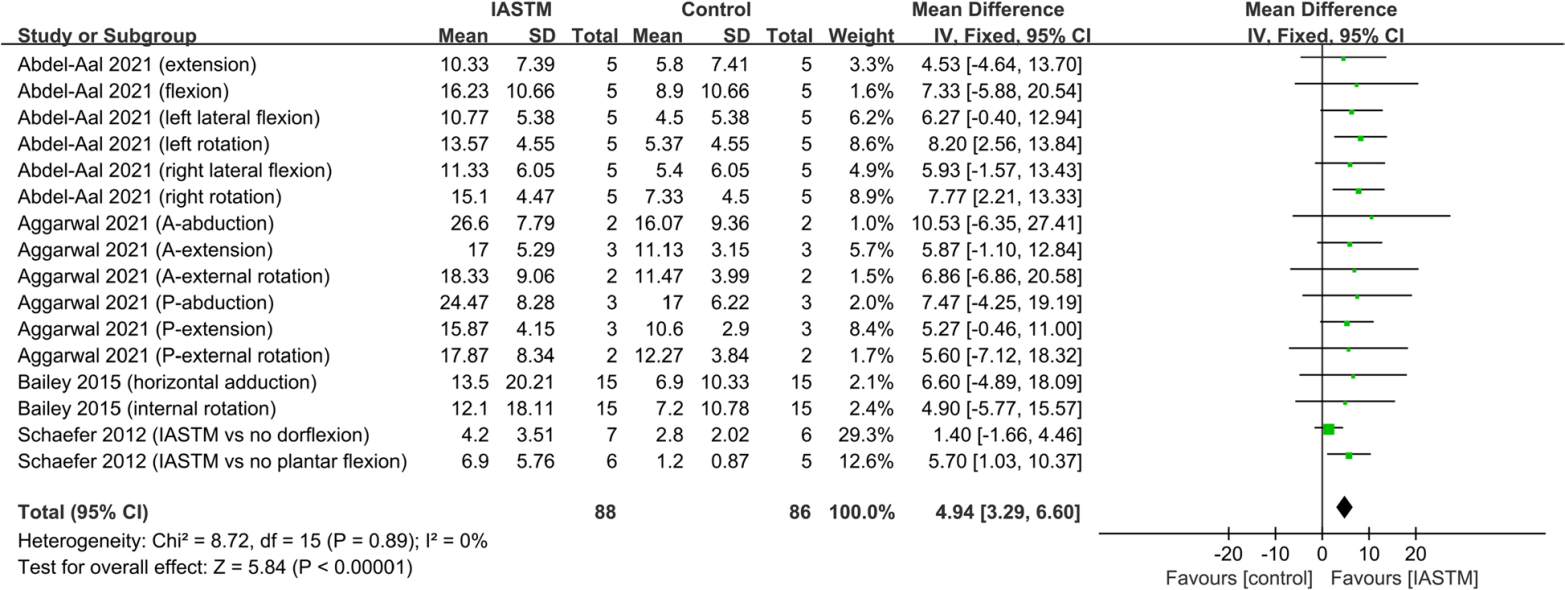
Forest plot of the effect of IASTM on ROM in ROM deficits individuals (in degree)

Considering that both patients with musculoskeletal disorders and healthy people with ROM deficits have ROM limitations, we analyzed these two factors together. Collectively (trials=4), 88 participants were in the IASTM treatment group, and 86 participants were in the control group. All 4 studies compared IASTM plus other treatment(s) to other treatment(s) (two studies used conventional treatments as the other treatments, and the other two used stretching as the other treatment) [28, 29, 31, 37]. IASTM significantly improved ROM (MD = 4.94, 95% CI: 3.29 to 6.60, *p* < 0.00001, I^2^ = 0%) (Fig. 3). Sensitivity analyses showed stable results (see Additional file 3).

#### Effect of IASTM on ROM in healthy individuals without ROM deficits (in degree)

**Fig. 4:**
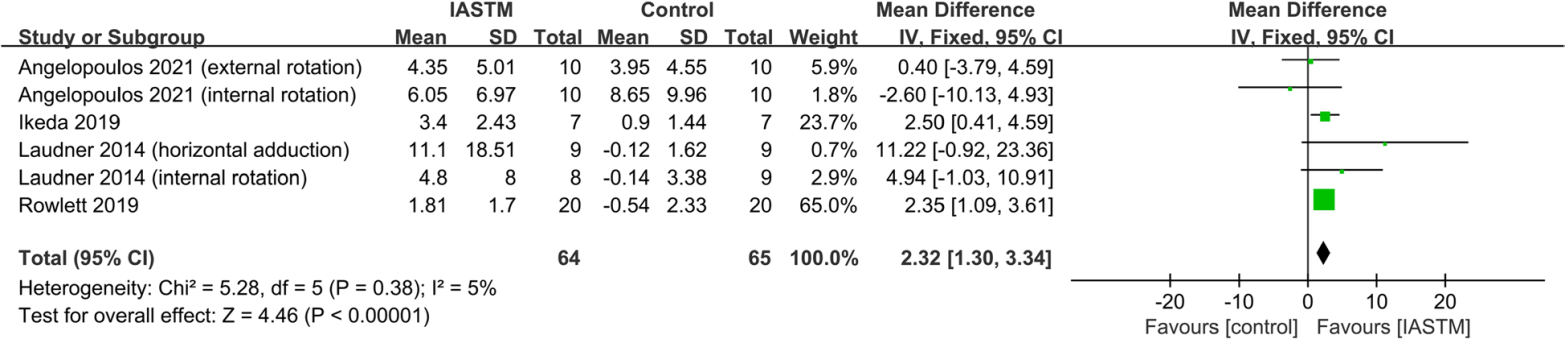
Forest plot of the effect of IASTM on ROM in ROM unlimited individuals (in degree)

Collectively (trials=4), 64 participants were in the IASTM treatment group, and 65 participants were in the control group. IASTM significantly improved ROM (MD = 2.32, 95% CI: 1.30 to 3.34, *p* < 0.00001, I^2^ = 5%) (Fig. 4). Sensitivity analyses showed stable results (see Additional file 4). Of the 4 studies, two compared IASTM alone with controls, while the other two compared IASTM plus other treatments with other treatments (the other treatments were kinetic flossing and step taps) [30, 33, 34, 36]. The subgroup analyses indicated that IASTM could significantly improve ROM when IASTM alone was used (MD = 2.99, 95% CI: 1.04 to 4.93, *p* = 0.003, I^2^ = 16%) or when combined therapies were used (MD = 2.07, 95% CI: 0.87 to 3.26, *p* = 0.0007, I^2^ = 12%) (see Additional file 5).

#### Effect of IASTM on ROM in healthy individuals with ROM deficits (in centimeter)

**Fig. 5:**
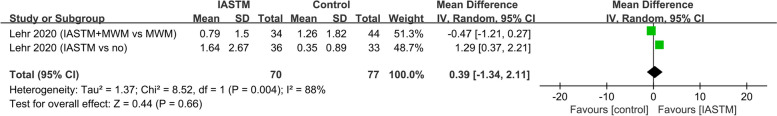
Forest plot of the effect of IASTM on ROM in ROM deficits individuals (in centimeter)

Collectively (trials=1), 70 participants were in the IASTM treatment group, and 77 participants were in the control group. The pooled results indicated that IASTM could not improve ROM (MD = 0.39, 95% CI: -1.34 to 2.11, *p* = 0.66, I^2^ = 88%) (Fig. 5).

## Discussion

The results of our study showed that IASTM could improve ROM in degree in healthy individuals with or without ROM deficits, or in patients with musculoskeletal disorders.

In recent years, researchers have investigated the impact of IASTM on ROM from various angles. Cheatham et al. [15] conducted an online survey of 853 members of the National Athletic Trainers' Association and the American Physical Therapy Association and found that the majority of respondents believed that IASTM improved ROM. Brandl et al. [38] reported that the bioimpedance of tissues increases after IASTM, suggesting that IASTM reduces the water content of tissues. Then, the tissue may gain more water through a delayed supercompensatory effect [39], thereby increasing the flexibility of the tissue. The results of these two studies, as well as our results in degree, indicated that IASTM improves ROM. However, we only had very low to low certainty based on the Grading of Recommendations Assessment, Development, and Evaluation scores [40], with downgrading for study limitations, imprecision, and publication bias. As a result, more high-quality randomized controlled studies are needed in the future.

To date, two meta-analyses have investigated the impact of IASTM on ROM [22, 23]. Both studies reported that IASTM did not improve ROM [22, 23], which contrasts with our results in degree. This discrepancy may be attributed to the use of distinct inclusion and exclusion criteria, and effect indicators. Previous meta-analyses included studies comparing IASTM with other treatments or placebo and found no significant difference between the two by combining the data as a basis for the conclusion that IASTM did not improve ROM [22, 23]. However, the possibility that both interventions were effective was ignored. We included only studies comparing IASTM with controls and IASTM plus other treatments with other treatments, and the combined results in degree merging both supported IASTM, with a significant difference in *p*-values. Previous studies have also shown that some of the results of the included studies presented significant differences in the *P*value, but instead of basing the efficacy judgment on these results, the authors further compared the increase in ROM with the minimum clinically important difference and found that the changes did not reach the threshold, therefore, they concluded that IASTM was unable to improve ROM [22, 23]. In contrast, we used *P* values to assess the efficacy of the interventions because the included participants included individuals without ROM deficits. In addition, we excluded one negative study [21], which was included in both previous studies [22, 23]. The reason for exclusion was that we considered the randomization described in the text to be nonrandom. Therefore, previous studies may have underestimated the validity of IASTM, but our results were more accurate. Additionally, we included more studies (comparing IASTM alone to controls and IASTM plus other treatments to other treatments) and the quality of the included studies was higher than the quality of the included studies in the two previous studies (one study in our study was rated as low risk, while all the included studies were rated as high risk in the previous meta-analyses [22, 23]), which also increased the credibility of our results.

To our surprise, the results in centimeter showed that IASTM failed to improve ROM. The two sets of data were derived from the same study, in which IASTM alone was effective and combined therapies were ineffective. The authors of this study suggested that the results may stem from overloaded neurophysiological thresholds, which are exceeded by the combination treatment, diminishing the benefit of the treatment [35]. However, it is difficult to explain the results of our subgroup analyses among healthy individuals without ROM deficits, in which both IASTM alone and combined therapies were effective. Superficially, the two opposite results in our study seem to be caused by the different units of measurement. However, we still think that the more likely reason is the limited number of included studies. More randomized controlled studies in centimeter (including those at low risk) are needed in the future to assess the validity of IASTM on ROM and to explore the sources of heterogeneity.

This study has several limitations. First, only a few studies and participants were included, resulting in the inability to reach a definitive conclusion (including judging publication bias). Second, we lack sufficient data to perform independent analyses of combined therapies and IASTM alone, and we lack adequate data to analyze the effects of different treatment durations on treatment outcomes. Third, only the outcome at the end of the treatment was utilized, with no consideration given to intermediate measurements or those taken during follow-up. Fourth, we merged two datasets from the same study due to the scarcity of studies, potentially compromising the independence principle in meta-analyses. Fifth, we split the sample size in some studies, which would change the weights of these studies in the evidence synthesis. Finally, several deviations from the original protocol were made during this study. We have updated the search date and expanded the literature search to cover all possible articles that met our study criteria. We also conducted unplanned subgroup analyses.

## Conclusions

IASTM can improve ROM in degree in healthy individuals with or without ROM deficits, or in patients with musculoskeletal disorders (with very low to low certainty). More high-quality studies (including different units) are needed in the future to explore the effects of IASTM on ROM.

### Supplementary Information

**Supplementary Material 1.** 

**Supplementary Material 2.** 

**Supplementary Material 3.** 

**Supplementary Material 4.** 

**Supplementary Material 5.** 

## Data Availability

All data generated or analyzed during this study are included in this published article and its supplementary information files.

## Abbreviations

CI: Confidence interval
IASTM: Instrument-assisted soft tissue mobilization
KT: Kinesiology taping
MD: Mean difference
MeSH: Medical subject heading
MWM: Mobilization with movement
ROM: Range of motion
PRISMA: Preferred Reporting Items for Systematic Reviews and Meta-Analyses
SD: Standard deviation
