# Supplementary material for: The effectiveness of instrument-assisted soft tissue mobilization on range of motion: a meta-analysis
Source: BMC Musculoskelet Disord. 2024 Apr 23;25:319. doi: 10.1186/s12891-024-07452-8 (PMC11036573; doi:10.1186/s12891-024-07452-8)
Supplement: Supplementary file 1 — Supplementary Material 1. [file 12891_2024_7452_MOESM1_ESM.docx]

**Additional file 1** The search strategies

The search strategy used in **PubMed**: from inception to 23 December 2023

((“instrument assisted soft tissue mobilization” [All Fields]) OR (“soft tissue mobilization technique” [All Fields]) OR (“instrument assisted soft tissue treatment technique” [All Fields]) OR (“instrument assisted soft tissue technique” [All Fields]) OR (“instrument assisted cross fiber massage” [All Fields]) OR (“augmented soft tissue mobilization” [All Fields]) OR (“IASTM” [All Fields]) OR (“IASTT” [All Fields]) OR (“IACFM” [All Fields]) OR (“ASTM” [All Fields]) OR (“ISTM” [All Fields]) OR (“Graston” [All Fields]) OR (“Ergon” [All Fields]) OR (“ASTYM” [All Fields]) OR (“Gua Sha” [All Fields]) OR (“instrument assisted” [All Fields]) OR (“soft tissue mobilization” [All Fields]) OR (“soft tissue manipulation” [All Fields]) OR **("Musculoskeletal Manipulations"[Mesh]) OR ("Musculoskeletal Manipulation"**[All Fields]) OR (“Manipulations, Musculoskeletal” [All Fields]) OR (“Manipulation Therapy” [All Fields]) OR (“Manipulative Therapies” [All Fields]) OR (“Manipulative Therapy” [All Fields]) OR (“Therapies, Manipulative” [All Fields]) OR (“Therapy, Manipulative” [All Fields]) OR (“Therapy, Manipulation” [All Fields]) OR (“Manipulation Therapies” [All Fields]) OR (“Therapies, Manipulation” [All Fields]) OR (“Reflexology” [All Fields]) OR (“Bodywork” [All Fields]) OR (“Bodyworks” [All Fields]) OR (“Rolfing” [All Fields]) OR (“Craniosacral Massage” [All Fields]) OR (“Massage, Craniosacral” [All Fields]) OR (“Manual Therapies” [All Fields]) OR (“Manual Therapy” [All Fields]) OR (“Therapies, Manual” [All Fields]) OR (“Therapy, Manual” [All Fields])) AND ((“Range of Motion, Articular”[Mesh]) OR (“Joint Range of Motion” [All Fields]) OR (“Joint Flexibility” [All Fields]) OR (“Flexibility,joint” [All Fields]) OR (“Range of Motion” [All Fields]) OR (“Passive Range of Motion” [All Fields]))

The search strategy used in **Web of Science**: from inception to 23 December 2023

ALL=(“instrument assisted soft tissue mobilization” OR “soft tissue mobilization technique” OR “instrument assisted soft tissue treatment technique” OR “instrument assisted soft tissue technique” OR “instrument assisted cross fiber massage” OR “augmented soft tissue mobilization” OR “IASTM” OR “IASTT” OR “IACFM” OR “ASTM” OR “ISTM” OR “Graston” OR “Ergon” OR “ASTYM” OR “Gua Sha” OR “instrument assisted” OR “soft tissue mobilization” OR “soft tissue manipulation” OR **"Musculoskeletal Manipulations" OR "Musculoskeletal Manipulation"** OR “Manipulations, Musculoskeletal” OR “Manipulation Therapy” OR “Manipulative Therapies” OR “Manipulative Therapy” OR “Therapies, Manipulative” OR “Therapy, Manipulative” OR “Therapy, Manipulation” OR “Manipulation Therapies” OR “Therapies, Manipulation” OR “Reflexology” OR “Bodywork” OR “Bodyworks” OR “Rolfing” OR “Craniosacral Massage” OR “Massage, Craniosacral” OR “Manual Therapies” OR “Manual Therapy” OR “Therapies, Manual” OR “Therapy, Manual”) AND ALL=(“Range of Motion, Articular” OR “Joint Range of Motion” OR “Joint Flexibility” OR “Flexibility,joint” OR “Range of Motion” OR “Passive Range of Motion”)

The search strategy used in **Embase**: from inception to 23 December 2023

(“instrument assisted soft tissue mobilization” OR “soft tissue mobilization technique” OR “instrument assisted soft tissue treatment technique” OR “instrument assisted soft tissue technique” OR “instrument assisted cross fiber massage” OR “augmented soft tissue mobilization” OR “IASTM” OR “IASTT” OR “IACFM” OR “ASTM” OR “ISTM” OR “Graston” OR “Ergon” OR “ASTYM” OR “Gua Sha” OR “instrument assisted” OR “soft tissue mobilization” OR “soft tissue manipulation” OR **"Musculoskeletal Manipulation"/syn OR "Musculoskeletal Manipulations"** OR “Manipulations, Musculoskeletal” OR “Manipulation Therapy” OR “Manipulative Therapies” OR “Manipulative Therapy” OR “Therapies, Manipulative” OR “Therapy, Manipulative” OR “Therapy, Manipulation” OR “Manipulation Therapies” OR “Therapies, Manipulation” OR “Reflexology” OR “Bodywork” OR “Bodyworks” OR “Rolfing” OR “Craniosacral Massage” OR “Massage, Craniosacral” OR “Manual Therapies” OR “Manual Therapy” OR “Therapies, Manual” OR “Therapy, Manual”) AND (“Range of Motion”/syn OR “Range of Motion, Articular” OR “Joint Range of Motion” OR “Joint Flexibility” OR “Flexibility,joint” OR “Passive Range of Motion”)

The search strategy used in **The Cochrane library**: from inception to 23 December 2023

[All Text] #1 “instrument assisted soft tissue mobilization” OR “soft tissue mobilization technique” OR “instrument assisted soft tissue treatment technique” OR “instrument assisted soft tissue technique” OR “instrument assisted cross fiber massage” OR “augmented soft tissue mobilization” OR “IASTM” OR “IASTT” OR “IACFM” OR “ASTM” OR “ISTM” OR “Graston” OR “Ergon” OR “ASTYM” OR “Gua Sha” OR “instrument assisted” OR “soft tissue mobilization” OR “soft tissue manipulation”

[Mesh] #2 **“Musculoskeletal Manipulations”**

[All Text] #3 **"Musculoskeletal Manipulation"** OR “Manipulations, Musculoskeletal” OR “Manipulation Therapy” OR “Manipulative Therapies” OR “Manipulative Therapy” OR “Therapies, Manipulative” OR “Therapy, Manipulative” OR “Therapy, Manipulation” OR “Manipulation Therapies” OR “Therapies, Manipulation” OR “Reflexology” OR “Bodywork” OR “Bodyworks” OR “Rolfing” OR “Craniosacral Massage” OR “Massage, Craniosacral” OR “Manual Therapies” OR “Manual Therapy” OR “Therapies, Manual” OR “Therapy, Manual”

#4 #1 OR #2 OR #3

[Mesh] #5 “Range of Motion, Articular”

[All Text] #6 “Joint Range of Motion” OR “Joint Flexibility” OR “Flexibility,joint” OR “Range of Motion” OR “Passive Range of Motion”

#7 #5 or #6

#8 #4 and #7
