# Supplementary material for: The effectiveness of instrument-assisted soft tissue mobilization on range of motion: a meta-analysis
Source: BMC Musculoskelet Disord. 2024 Apr 23;25:319. doi: 10.1186/s12891-024-07452-8 (PMC11036573; doi:10.1186/s12891-024-07452-8)
Supplement: Supplementary file 2 — Supplementary Material 2. [file 12891_2024_7452_MOESM2_ESM.docx]

**Additional file 2** Detailed assessment of bias risk

| Bias | Authors’ judgment | Support for judgment |
| --- | --- | --- |
| Schaefer and Sandrey (2012) ^37^ |  |  |
| Random sequence generation | Unclear | The paper describes participants were randomly grouped and matched according to age, height, injured ankle, but does not describe the details of generating the random sequence |
| Allocation concealment | Unclear | The paper mentions that allocation was concealed, but how to conceal was not described |
| Blinding of participants and personnel | High | The blindness of the therapist and patients were not attainable due to the nature of the intervention |
| Blinding of outcome assessment | High | The same researcher did all the treatment, testing and training sessions |
| Incomplete outcome data | High | 2/15 (13.33%) lost in the DBT and IASTM group, 3/15 (20%) lost in the DBT and Sham IASTM group, 4/15 (26.67%) lost in the DBT only group |
| Selective reporting | Low | All relevant outcomes were described |
| Other bias | Low | Unsuspectd predatory journal |
| Laudner et al (2014) ^34^ |  |  |
| Random sequence generation | Unclear | The paper mentions the use of random grouping, but does not provide any details |
| Allocation concealment | Unclear | No description |
| Blinding of participants and personnel | High | The blindness of the therapist and patients were not attainable due to the nature of the intervention |
| Blinding of outcome assessment | Low | Before intervention, participants were lying in a supine position with their shirts on, and two investigators did the pretest measurement. After the test, both investigators left the room. Then, the participants removed their shirts and lay down in a prone position and another investigator did the treatment. After finishing, the participants put their shirts back on and returned to the original supine position to hide erythema that may have been caused by treatment. The previous investigator returned to the room and did posttest measurement. |
| Incomplete outcome data | Low | No participant lost to be thought for only one treatment, although the number of participants at the posttest measurement is not described in the paper |
| Selective reporting | Low | All relevant outcomes were described |

(Continued)

**Additional file 2** (Continued)

| Bias | Authors’ judgment | Support for judgment |
| --- | --- | --- |
| Other bias | Low | Unsuspectd predatory journal |
| Bailey et al (2015) ^31^ |  |  |
| Random sequence generation | Low | Participants were randomly assigned by drawing |
| Allocation concealment | Unclear | The paper mentions that the primary investigator was blinded to group assignment, but does not provide any details |
| Blinding of participants and personnel | High | The blindness of the therapist and patients were not attainable due to the nature of the intervention |
| Blinding of outcome assessment | Low | The investigator who was blinded to group assignment was absent while treatment was done. In addition, all participants were positioned supine to blind the investigator to any potential erythema associated with the treatment |
| Incomplete outcome data | Low | No participant lost to be thought for only one treatment, although the number of participants at the posttest measurement is not described in the paper |
| Selective reporting | Low | All relevant outcomes were described |
| Other bias | Low | Unsuspectd predatory journal |
| Ikeda et al (2019) ^33^ |  |  |
| Random sequence generation | Unclear | The abstract mentions that this is a randomized study, but there is no relevant description in the main text |
| Allocation concealment | Unclear | No description |
| Blinding of participants and personnel | High | The blindness of the therapist and patients were not attainable due to the nature of the intervention |
| Blinding of outcome assessment | Unclear | No description |
| Incomplete outcome data | Unclear | No description |
| Selective reporting | Low | All relevant outcomes were described |
| Other bias | Low | Unsuspectd predatory journal |
| Rowlett et al (2019) ^36^ |  |  |

(Continued)

**Additional file 2** (Continued)

| Bias | Authors’ judgment | Support for judgment |
| --- | --- | --- |
| Random sequence generation | Low | Participants were randomly assigned by opaque envelope |
| Allocation concealment | Unclear | The paper mentions that the first researcher was blinded to group assignment, but does not provide any details |
| Blinding of participants and personnel | High | The blindness of the therapist and patients were not attainable due to the nature of the intervention |
| Blinding of outcome assessment | Low | The investigator did pretest measurements. Then, the participants were directed to another room and recived treatment by another researcher. Following intervention, posttest measurements was reassessed by the initial investigator. In addition, the paper mentions that the investigator was blinded to the intervention delivered. |
| Incomplete outcome data | Low | No participant lost to be thought for only one treatment, although the number of participants at the posttest measurement is not described in the paper |
| Selective reporting | Low | All relevant outcomes were described |
| Other bias | Low | Unsuspectd predatory journal |
| Abdel-Aal et al (2021) ^28^ |  |  |
| Random sequence generation | Low | Participants were randomly assigned according to computer-generated block randomization |
| Allocation concealment  Blinding of participants and personnel  Blinding of outcome assessment  Incomplete outcome data  Selective reporting  Other bias | Low  High  Low  Low  Low  Low | Sealed, sequentially numbered opaque envelopes were used to ensure the concealed allocation  The blindness of the therapist and patients were not attainable due to the nature of the intervention  The first researcher who was blinded to the group assignment collected the outcome measures  No participants lost after two weeks inervation. 3/30 (10%) lost in the experimental group, 1/30 (3.3%) lost in the control group after four weeks intervation. In addition, intention to treat analysis with the multiple imputation model was applied for the missing data from the 4-weeks follow-up measurement  All relevant outcomes were described  Unsuspectd predatory journal |

(Continued)

**Additional file 2** (Continued)

| Bias | Authors’ judgment | Support for judgment |
| --- | --- | --- |
| Aggarwal et al (2021) ^29^ |  |  |
| Random sequence generation | Low | Participants were randomly assigned by a lottery method |
| Allocation concealment | Unclear | No description |
| Blinding of participants and personnel | High | The blindness of the therapist and patients were not attainable due to the nature of the intervention |
| Blinding of outcome assessment | Unclear | No description |
| Incomplete outcome data | Unclear | No description |
| Selective reporting | Low | All relevant outcomes were described |
| Other bias | Low | Unsuspectd predatory journal |
| Angelopoulos et al (2021) ^30^ |  |  |
| Random sequence generation | Unclear | The paper mentions the use of random grouping, but does not provide any details |
| Allocation concealment | Unclear | No description |
| Blinding of participants and personnel | High | The blindness of the therapist and patients were not attainable due to the nature of the intervention |
| Blinding of outcome assessment | Unclear | No description |
| Incomplete outcome data  Selective reporting  Other bias  Lehr et al (2022) ^35^  Random sequence generation  Allocation concealment  Blinding of participants and personnel  Blinding of outcome assessment | Low  Low  Low  Unclear  Unclear  High  Unclear | No participant lost to be thought for only one treatment, although the number of participants at the posttest measurement is not described in the paper  All relevant outcomes were described  Unsuspectd predatory journal  The paper mentions the use of random grouping, but does not provide any details  The paper mentions that the examiners were blinded to random assignment, but does not provide any details  The blindness of the therapist and patients were not attainable due to the nature of the intervention  The paper mentions that the physical therapists who performed the intervention were blinded to |

(Continued)

**Additional file 2** (Continued)

| Bias | Authors’ judgment | Support for judgment |
| --- | --- | --- |
|  |  | random post test results, but does not provide any details of outcome assessment |
| Incomplete outcome data | Low | No participant lost to be thought for only one treatment, although the number of participants at the posttest measurement is not described in the paper |
| Selective reporting | Low | All relevant outcomes were described |
| Other bias | Low | Unsuspectd predatory journal |
