# Supplementary figures and images for: The effectiveness of instrument-assisted soft tissue mobilization on range of motion: a meta-analysis
Source: BMC Musculoskelet Disord. 2024 Apr 23;25:319. doi: 10.1186/s12891-024-07452-8 (PMC11036573; doi:10.1186/s12891-024-07452-8)

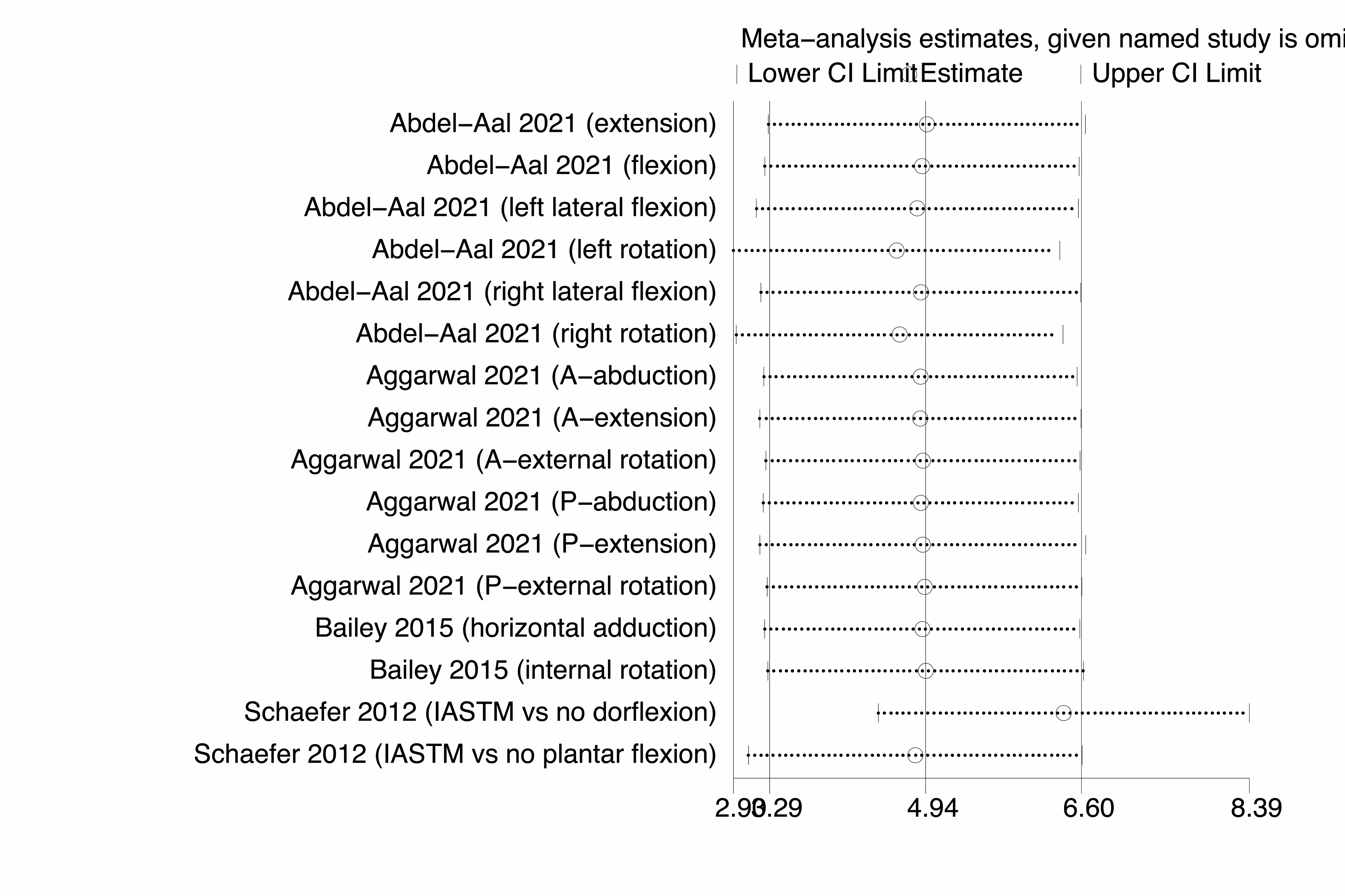


**Figure A 3** Sensitivity analysis of IASTM on ROM in individuals with ROM deficits (in degree)

Supplement: Supplementary file 3 — Supplementary Material 3. [file 12891_2024_7452_MOESM3_ESM.docx]

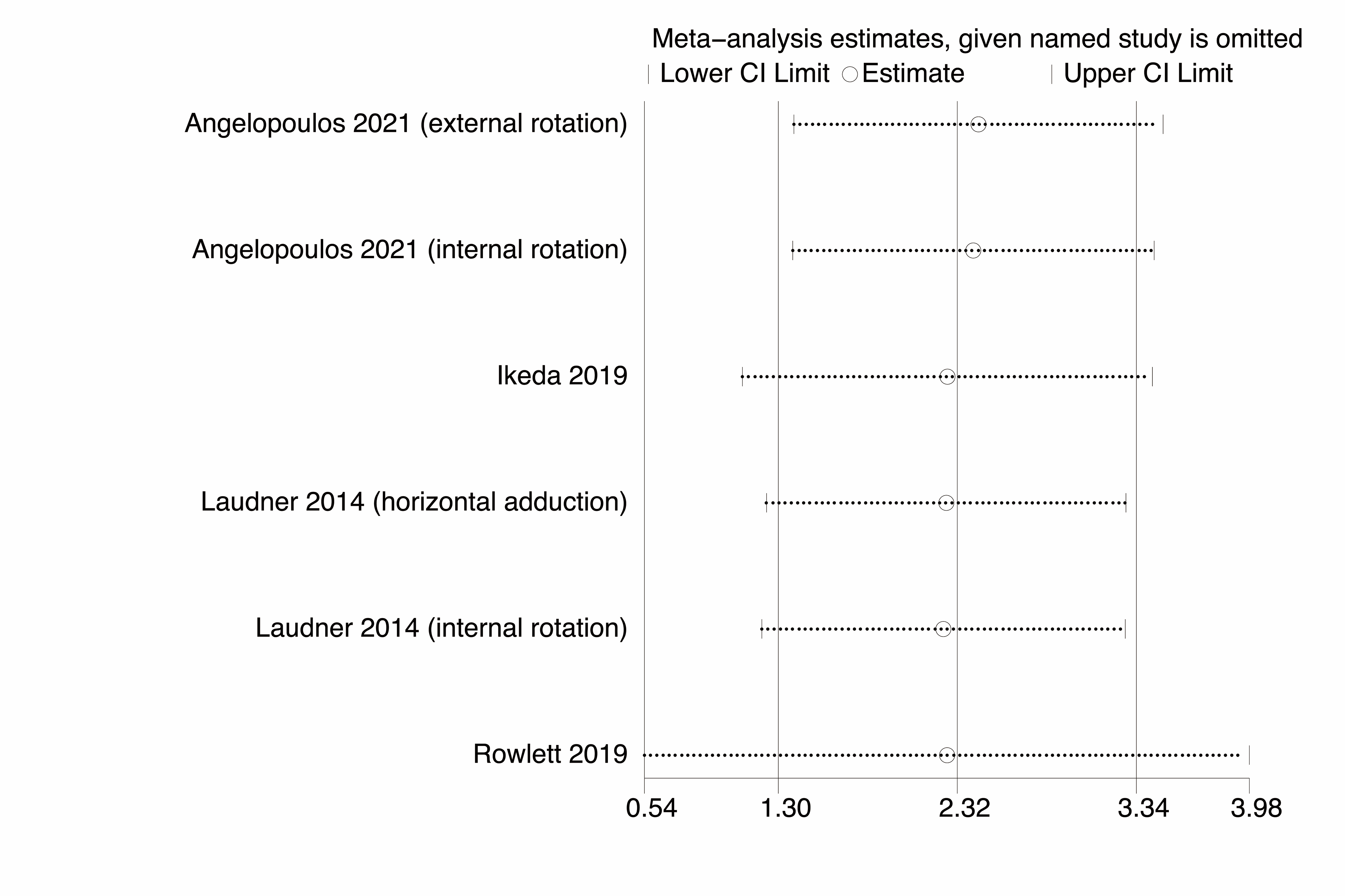


**Figure A 4** Sensitivity analysis of IASTM on ROM in individuals without ROM deficits (in degree)

Supplement: Supplementary file 4 — Supplementary Material 4. [file 12891_2024_7452_MOESM4_ESM.docx]

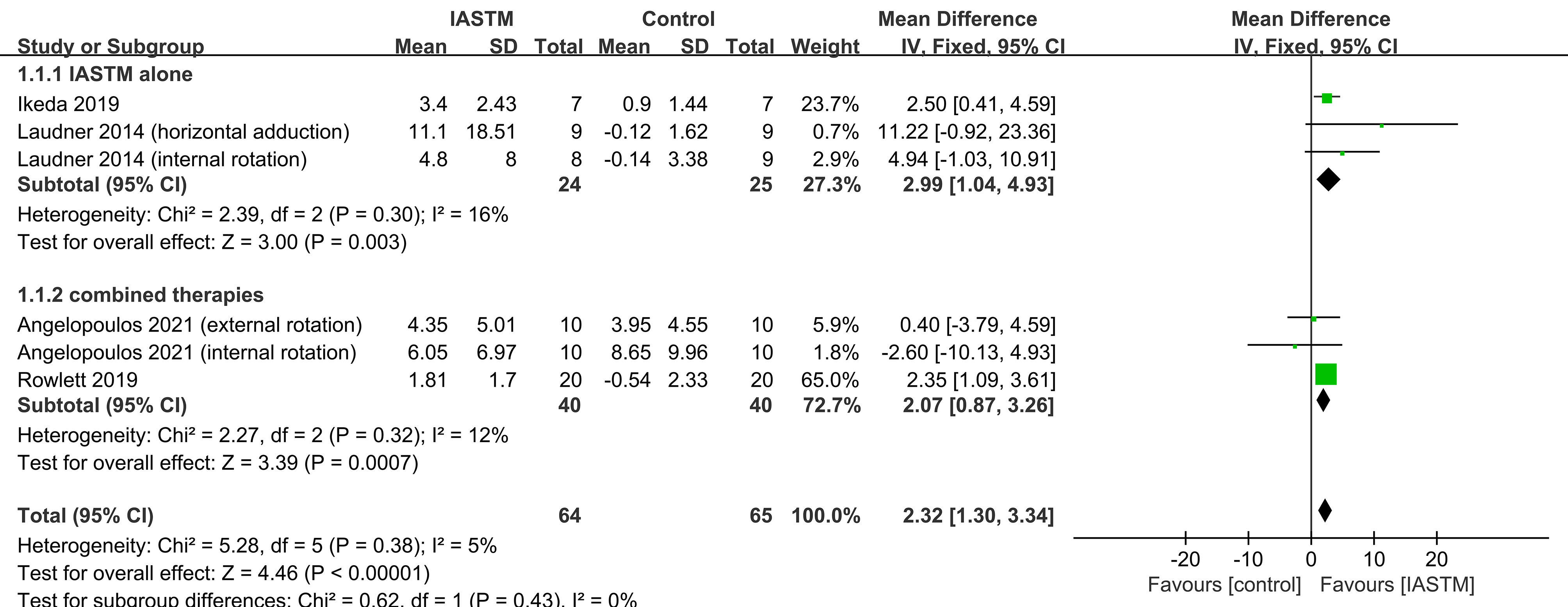


**Figure A 5** Forest plot of the subgroup analysis of different intervention methods (in degree)

Supplement: Supplementary file 5 — Supplementary Material 5. [file 12891_2024_7452_MOESM5_ESM.docx]
